# MLL Becomes Functional through Intra-Molecular Interaction Not by Proteolytic Processing

**DOI:** 10.1371/journal.pone.0073649

**Published:** 2013-09-10

**Authors:** Akihiko Yokoyama, Francesca Ficara, Mark J. Murphy, Christian Meisel, Chikako Hatanaka, Issay Kitabayashi, Michael L. Cleary

**Affiliations:** 1 Laboratory for Malignancy Control Research, Kyoto University Graduate School of Medicine, Kyoto, Japan; 2 Milan Unit, Istituto di Ricerca Genetica e Biomedica, Consiglio Nazionale delle Ricerche, Milan, Italy; 3 Humanitas Clinical and Research Center, Rozzano, Italy; 4 Department of Pathology, Stanford University School of Medicine, Stanford, California, United States of America; 5 Depertment of Neurology, University Clinic Carl Gustav Craus, Dresden, Germany; 6 Division of Hematological Malignancy, National Cancer Center Research Institute, Tokyo, Japan; Kanazawa University, Japan

## Abstract

The mixed lineage leukemia (MLL) protein is an epigenetic transcriptional regulator that controls proliferative expansion of immature hematopoietic progenitors, whose aberrant activation triggers leukemogenesis. A mature MLL protein is produced by formation of an intra-molecular complex and proteolytic cleavage. However the biological significance of these two post-transcriptional events remains unclear. To address their in vivo roles, mouse mutant alleles were created that exclusively express either a variant protein incapable of intra-molecular interaction (designated *de*) or an uncleavable mutant protein (designated *uc*). The *de* homozygous mice died during midgestation and manifested devastating failure in embryonic development and reduced numbers of hematopoietic progenitors, whereas *uc* homozygous mice displayed no apparent defects. Expression of MLL target genes was severely impaired in *de* homozygous fibroblasts but unaffected in *uc* homozygous fibroblasts. These results unequivocally demonstrate that intra-molecular complex formation is a crucial maturation step whereas proteolytic cleavage is dispensable for MLL-dependent gene activation and proliferation in vivo.

## Introduction

MLL (also known as MLL1, HRX and KMT2A) is an epigenetic transcriptional regulator that serves essential roles in embryonic and hematopoietic development. During embryogenesis, MLL maintains expression of Homeobox (*HOX*) genes to confer cellular identities along the anterior-posterior body axis [1], [2]. In the hematopoietic lineage, MLL regulates expression of a subset of *HOX* genes [3], [4] that promotes self-renewal of hematopoietic stem cells (HSCs) and expansion of immature progenitor pools [5], [6]. Hence, *Mll* deficiency in mice causes hematopoietic failure accompanied with insufficient expansion of immature hematopoietic progenitors [3], [7], [8]. Furthermore, MLL suppresses premature-senescence in both human and mouse fibroblasts in part by maintaining *HOX* gene expression [8], [9], [10]. Therefore loss of MLL function causes premature senescence. Conversely, *MLL* gain-of-function mutations caused by chromosomal translocations in hematopoietic cells result in constitutive expression of *HOX* genes that aberrantly enhance proliferation [11], [12] and suppress senescence to cause acute leukemia [8], [10].

MLL is translated as a large precursor protein (430 kD) that subsequently undergoes proteolytic processing into two fragments (MLL^N^ and MLL^C^) by the Taspase 1 endopeptidase [13], [14], [15], which specifically cleaves sites that are evolutionally conserved with MLL2 (also known as MLL4, HRX2 and KMT2B) and Drosophila TRX. The respective MLL^N^ and MLL^C^ fragments form a holocomplex by non-covalent intra-molecular interaction [14], [15]. Although these MLL fragments are susceptible to distinct degradation pathways [8], the MLL^N^/MLL^C^ holocomplex is stably expressed because intra-molecular complex formation masks structures that would otherwise lead to degradation. However, the biological significance of these maturation processes in vivo remains unclear.

In the current study, the in vivo roles of intra-molecular complex formation and proteolytic processing in MLL functions were examined using knock-in mouse lines with targeted mutations that selectively prevent self-association and proteolytic cleavage, respectively.

## Materials and Methods

### Ethics statement

All animal work has been conducted according to the institutional guidelines with the approval of Stanford University (9839) and National Cancer Center Research Institute (T08-030-N, T08-030-CB02).

### Generation of knock-in mice

Targeting vectors containing the mutations and the cassettes of Neomycin resistance gene (neo) and Diphtheria toxin gene (DT) (kindly provided by Dr. Takeshi Yagi) were constructed by PCR-mediated mutagenesis and restriction enzyme digestion/ligation. The targeting vector for the *uc* mutation was constructed in the same manner as the previously published *dC* mutant allele [8]. ES cells (CGR8.8) were transfected with the linearized targeting vectors and screened for positive clones by PCR. Homologous recombination was confirmed by LA-PCR (Takara Bio Inc., Otsu, Japan) using primer pairs specific for both ends of the targeting construct (primer sequences available upon request). Targeted ES clones were transiently transfected with a Cre recombinase expression vector (kindly provided by Dr. Takeshi Yagi) and subsequently screened for clones with appropriate excision of the neo cassette. Blastocyst injections were performed by the Transgenic Research Facility of Stanford University. Knock-in mouse lines were maintained by backcrossing onto a C57BL/6 genetic background. Genotyping of mice for the *de* allele was performed by PCR using a primer set (5′- tgaactggtgggaaagcacagacatcctga -3′ and 5′-agagatggttcagcggttaagagctctgac-3′) that detected both the mutant allele (∼500 bp) and the wild type allele (800 bp). Genotyping of mice for the *uc* allele was performed by PCR using a primer set (5′-gttctgaagcacacattccacacc-3′ and 5′-catcaaagcgaagggcaatcagtg-3′) that detected both the mutant allele (∼310 bp) and the wild type allele (250 bp).

### Cell culture

293T, plat-E, and mouse embryonic fibroblast (MEF) cells were cultured in Dulbecco's modified Eagle's medium (DMEM) supplemented with 15% fetal calf serum and non-essential amino acids.

### Western blotting

Western blotting was performed as described previously [15]. The mouse monoclonal anti-MLL^N^ antibody (mmN4) and anti-MLL^C^ antibody (9–12) were previously described [8], [15]. Goat anti-menin antiserum (C19) was purchased from Santa Cruz Biotechnology Inc (Santa Cruz, CA) and mouse anti-actin antibody (MAB 1501R) was purchased from Millipore (Billerica, MA).

### RT-PCR

Reverse transcription (RT) was performed as described previously [8]. RT-PCR of the mouse *de* variant transcript was performed using a primer set flanking the exon 11 counter part sequences (5′-agatggagtccacaggatca-3′ and 5′-tttcttcgtgggtttggtgg-3′). Quantitative PCR (qPCR) was performed in triplicate and average expression levels (with standard deviations) normalized to that of *Gapdh* or *Actb* were calculated using a standard curve and the relative quantification method as described in ABI User Bulletin #2. Taqman probes for various genes [*Gapdh*:Mm99999915_g1, *Actb*:Mm00607939_s1, *Mll*(N):Mm0117926_g1, *Mll*(M):Mm01179218_m1, *Mll*(C):Mm10179235_m1, *Hoxc8*:Mm00439369_m1, *Cdkn2a*:Mm00494449_m1, *Cdkn1b*:Mm00438167_g1, *Cdkn2c*:Mm00483243_m1, *Hoxc4*:Mm00442838_m1, *Hoxc9*:Mm00433972_m1, *PAI-1*(*Serpine1*):Mm00435860_m1, *Men1*:Mm00484963_m1, *Ledgf* (*Psip1*):Mm01259222_g1, *Hoxa10*:Mm00433966_m1, *Hoxa9*:Mm00439364_m1, *Hoxa7*:Mm00657963_m1] were purchased from Applied Biosystems (Foster City, CA).

### Flow cytometry analysis and sorting

Flow cytometry was performed as previously described [8], [16]. Single cell suspensions harvested from the bone marrow and thymus were stained in deficient RPMI (Irvine Scientific, Santa Ana, CA) containing 3% fetal calf serum, 1 mM EDTA and 10 mM HEPES. Conjugated monoclonal antibodies (mAbs) were obtained from either BD (Flanklin Lakes, NJ) or eBioscience (San Diego, CA). The lineage cocktail included antibodies for Gr1 (RB6-8C5), B220 (RA3-6B2), TER119 (TER-119), CD3 (145-2C12), CD4 (GK1.5), and CD8 (53-6.7). The following mAbs were also used: Mac1/CD11b (M1/70), cKit (2B8), Sca1 (D7), CD48 (HM48-1), CD34 (49E8), CD16/32 (93), Flk2 (A2F10), CD45.2 (104), and CD43 (S7). Stained cells were analyzed with LSR-1A or LSR-II flow cytometer (BD). J-SAN (Bay bioscience, Kobe, JAPAN) was used for cell sorting. Cell Quest Pro or Diva (BD) was used for data acquisition, and FlowJo (Tree Star Inc., Ashland, OR) was used for analysis.

### *In vivo* reconstitution assay

Fetal liver cells of *de* homozygous mutant (5×10^5^ cells) or the wild type/heterozygous controls (5×10^4^ cells) harvested from E12.5 embryos or white blood cells harvested from adult *uc* homozygous mice (1×10^6^ cells) or the wild type control (1×10^6^ cells) in the littermates were injected intravenously into lethally irradiated (1200 rads in two days or 900 rads in one day) C57BL/6 mice. Recipient mice were maintained on water supplemented with antibiotics for a few weeks after transplantation.

### Whole mount in situ hybridization

In situ hybridization was performed on E10.5 embryos as described elsewhere [8], [17]. The *Hoxc8* probe was synthesized using DIG RNA labeling kit (Roche) and hybridized with pre-treated embryos. After washing, the probe was visualized by anti-digoxygenin antibody coupled with alkaline phosphatase. The plasmid for the *Hoxc8* probe was kindly provided by Dr. Licia Selleri.

### MEF proliferation and 3T3 senescence assays

MEFs were derived from E11.5 embryos and analyzed as described elsewhere [8], [18]. The MEFs were plated at the concentration of 10^4^ cells/ml on Day 0 and the cell count was measured at each time point. In 3T3 senescence assay, the MEFs were re-plated every 3 days.

### Myeloid progenitor serial replating assay

Myeloid progenitor serial replating assay was described elsewhere [19], [20]. Myeloid progenitor cells were harvested from the femurs of mice. C-kit positive cells were enriched by immuno-magnetic selection using an Auto MACS (Miltenyi Biotech), transduced with recombinant retrovirus by spinoculation, and plated in methylcellulose medium (M3231, Stemcell Technologies) containing SCF, IL-3, IL-6 and GM-CSF. The colony-forming units (CFUs) per 10^4^ plated cells were quantified after 5–7 d of culture and expressed as the average and standard deviation of at least triplicate determinations.

## Results

### Intra-molecular complex formation of MLL fragments is essential for MLL-dependent gene activation

**Figure 1:**
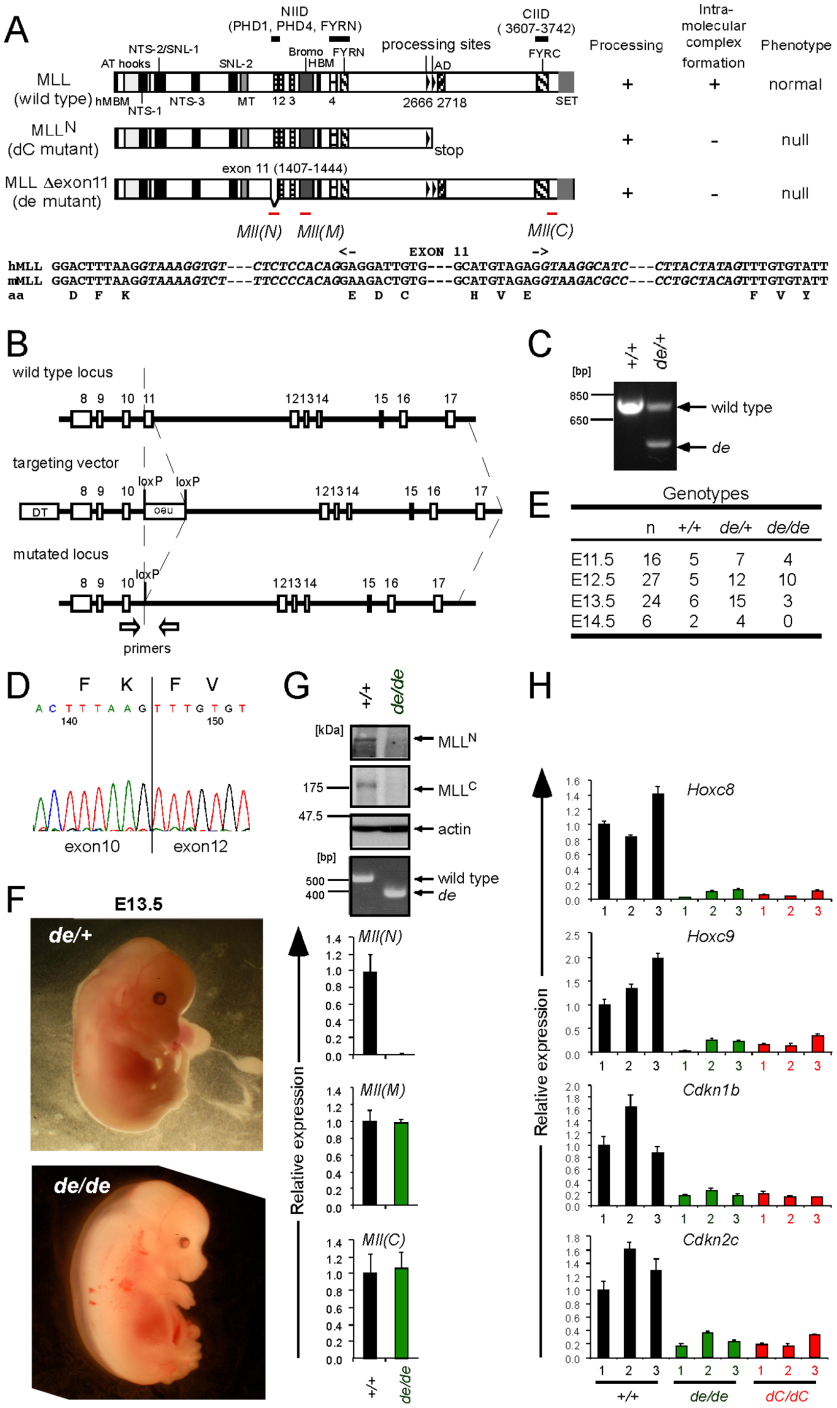
Intra-molecular interaction is required for MLL-dependent gene activation. A. MLL proteins produced by *dC* and *de* mutations are shown schematically. The characteristics of each mutant are shown on the right. The N-terminal intra-molecular interaction domain (NIID) includes PHD1, PHD4 and FYRN and the C-terminal intra-molecular interaction domain (CIID) is FYRC. Positions of the three taqman probes for *Mll* [*Mll*(N), *Mll*(M) and *Mll*(C)] used in Figure 1G are indicated by red bars. Genomic sequences around the exon 11 of the human MLL and its murine counterpart are shown at the bottom. B. Genomic structures of the wild type allele and the recombined allele are shown with the targeting vector. C. Diagnostic PCR identifies the recombined allele in genetically engineered mice. *+/+*: wild type. *de/+*: *de* heterozygous D. Sequence of the exon junction of *Mll de* transcript. The PCR product generated by RT-PCR of *de* homozygous MEFs was sequenced. E. Genotypes at various developmental stages. Viability of embryos was confirmed by presence of heart contractions. *de/de*: *de* homozygous F. Morphologies of E13.5 embryos of *de* mutants. G. Expression of MLL proteins and RNAs in MEFs. Expression of MLL^N^, MLL^C^, and actin was visualized by western blotting. Relative expression levels of *Mll* mRNAs (normalized to *Gapdh*) are expressed relative to those of wild type arbitrarily set as 1. RT-PCR analysis demonstrated expression of the *de* mutant mRNAs. qPCR using the *Mll*(M) and *Mll*(C) taqman probe demonstrated that *Mll* mRNAs were expressed at the same levels in wild type and *de* homozygous MEFs, whereas the *Mll*(N) taqman probe showed that the murine exon corresponding to the human exon 11 was not transcribed. Error bars represent the standard deviations of triplicate PCRs. H. Expression of MLL target genes in *de* homozygous MEFs. Wild type and *dC* homozygous MEFs were also analyzed for comparison. Relative expression levels of various MLL target genes (normalized to *Gapdh*) are expressed relative to those of wild type-1 arbitrarily set as 1. RT-qPCR demonstrates that expression of MLL target genes including *Hoxc8*, *Hoxc9*, *Cdkn1b*, *Cdkn2c* was impaired in *de* homozygous and *dC* homozygous MEFs. Error bars represent the standard deviations of triplicate PCRs. *dC/dC*: *dC* homozygous.

The intra-molecular interaction domains of MLL have been defined as PHD fingers 1 and 4 (PHD1 and PHD4), FYRN, and FYRC domains [8], [15] (Figure 1A). Most of PHD1 is encoded by exon 11 of MLL. It has been reported that an MLL variant protein lacking exon 11 sequences is dominantly expressed in some cases of acute lymphoid leukemia [21]. We previously reported that this variant protein can be transiently expressed and efficiently processed but is incapable of forming an MLL^N^/ MLL^C^ holocomplex [8]. To investigate the in vivo roles of intra-molecular complex formation, a mutant allele (designated *de*) that lacks the exon 11 counterpart of mouse was generated in ES cells (Figure 1B) and its successful recombination was confirmed by diagnostic genomic PCR (Figure 1C) and RT-PCR followed by sequencing (Figure 1D). Another mutant allele (designated *dC*), which served as a control for null mutation, was previously engineered to contain a stop codon at the second processing site [8], thereby exclusively expressing MLL^N^ due to the inability to translate downstream MLL^C^ sequences within the *Mll* mRNA (Figure 1A).

Unlike wild type and *de* heterozygous mice, *de* homozygous mice died during midgestation (Figure 1E) with a similar phenotype displayed by *dC* homozygous (*dC*/*dC*) mice [8], including subcutaneous edema and hemorrhage (Figure 1F). Mouse embryonic fibroblasts (MEFs) derived from *de* homozygous embryos expressed MLL proteins at low or undetectable levels whereas their respective mRNAs were expressed at normal levels (Figure 1G), confirming previous findings that MLL protein fragments are subjected to degradation if unable to self-associate in an intra-molecular complex [8], [14]. Consequently, expression of MLL target genes including *Hoxc8*, *Hoxc9*, *Cdkn1b* and *Cdkn2c* was severely reduced in *de* homozygous MEFs [22], [23] (Figure 1H). These results clearly demonstrate that intra-molecular complex formation is required for stable expression of MLL proteins and thus for MLL-dependent gene activation in vivo.

### Intra-molecular complex formation is required for expansion of hematopoietic progenitors

**Figure 2:**
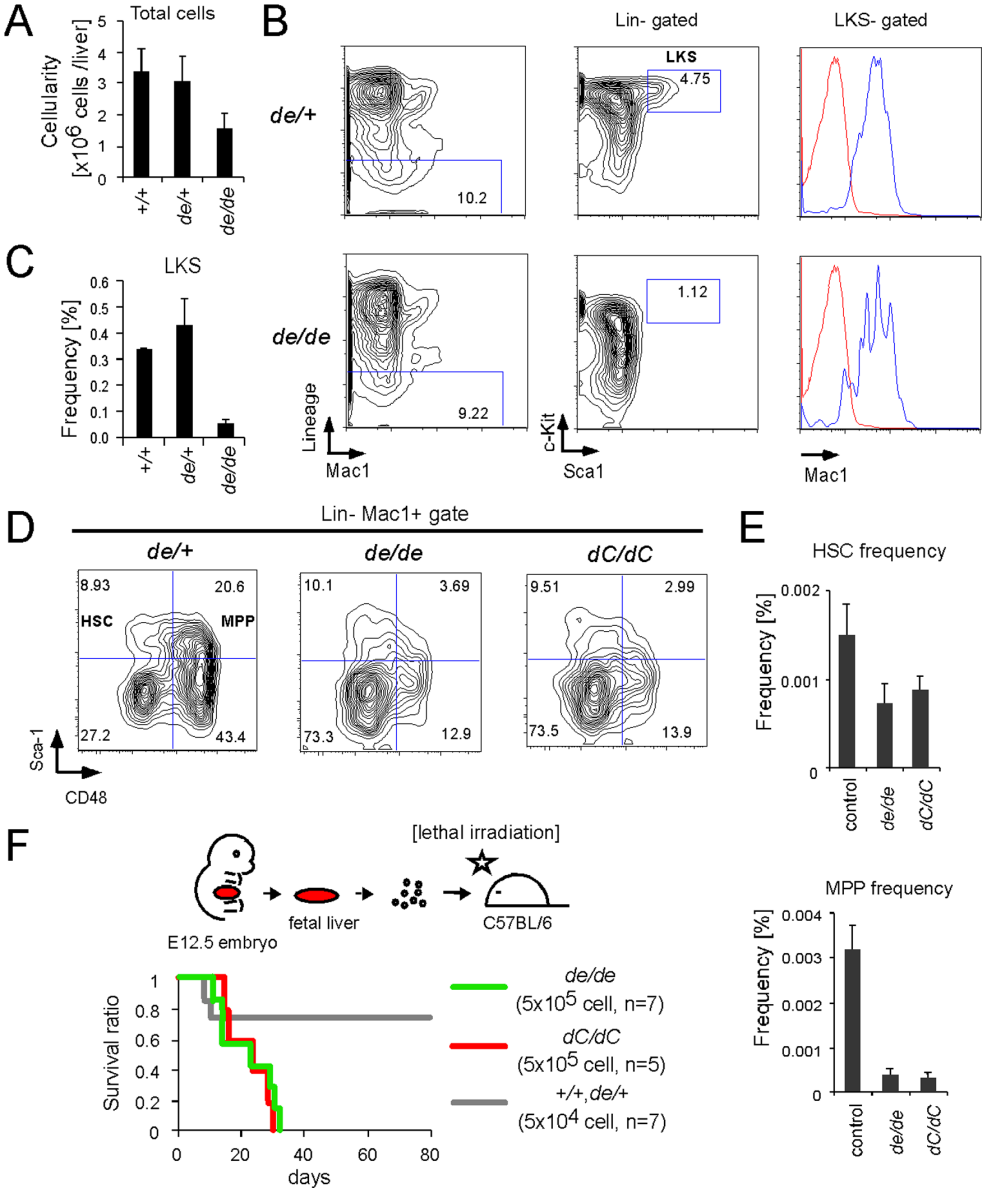
Intra-molecular interaction is required for expansion of hematopoietic progenitors. A. Cellularity of *de* mutant FLs. Error bars represent the standard deviations of cell numbers of FLs. For each genotype, at least 3 livers were analyzed. B. Population analysis of *^de^* mutant FLs by flow cytometry. The fetal LKS compartment was defined as the Lin^−^cKit^+^Sca-1^+^Mac1^+^ population. C. Frequency of LKS populations in *^de^* mutant FLs. Error bars represent the standard deviations of more than two littermate embryos for each genotype. D. Population analysis of HSCs from MPPs in *^de^* mutant FLs by flow cytometry. Lin^−^Mac1^+^ cells were subdivided by Sca-1 and CD48. E. Frequencies of HSCs (top) and MPPs (bottom) in *^de^* mutant FLs. Error bars represent the standard deviations of more than four embryos for each genotype. Frequency was expressed as the percentage of each fraction within the whole population. F. Ability of *^de^* mutant FL cells to reconstitute the hematopoietic system. 5×10^5^ cells of *^de^* homozygous FLs and 5×10^4^ cells of the wild type/heterozygous controls were transplanted into lethally irradiated syngenic mice. Previously published data for *^dC^* homozygous FLs [8] obtained in the same experimental setting were included for comparison. N: the number of FLs analyzed.

The effect of intra-molecular complex formation on proliferative expansion of hematopoietic progenitors was examined by flow cytometry analysis of the fetal livers (FLs) derived from *de* homozygous E12.5 embryos, which contained fewer hematopoietic cells compared to the wild type/heterozygous control FLs (Figure 2A). The LKS compartment, which contains HSCs and multi-potent progenitors (MPPs), was markedly reduced in *de* homozygous FLs (Figure 2B, 2C). Further analysis using the CD48 marker [24] showed that *de* homozygous FLs produced HSCs 50% less efficiently than the wild type/heterozygous controls (Figure 2D, 2E). MPP frequency was more profoundly affected by loss of intra-molecular complex formation, phenocopying *dC* homozygous FLs [8]. Transplantation of FL cells into lethally irradiated mice showed that *de* homozygous FL cells were unable to reconstitute the hematopoietic system, while a 10-fold lower cell number of the wild type/heterozygous control FL cells successfully reconstituted (Figure 2F). These results show that intra-molecular complex formation is essential for the functions of MLL to generate appropriate numbers of fetal HSCs and MPPs in vivo.

### Proteolytic processing of MLL is not required for MLL-dependent gene activation

**Figure 3:**
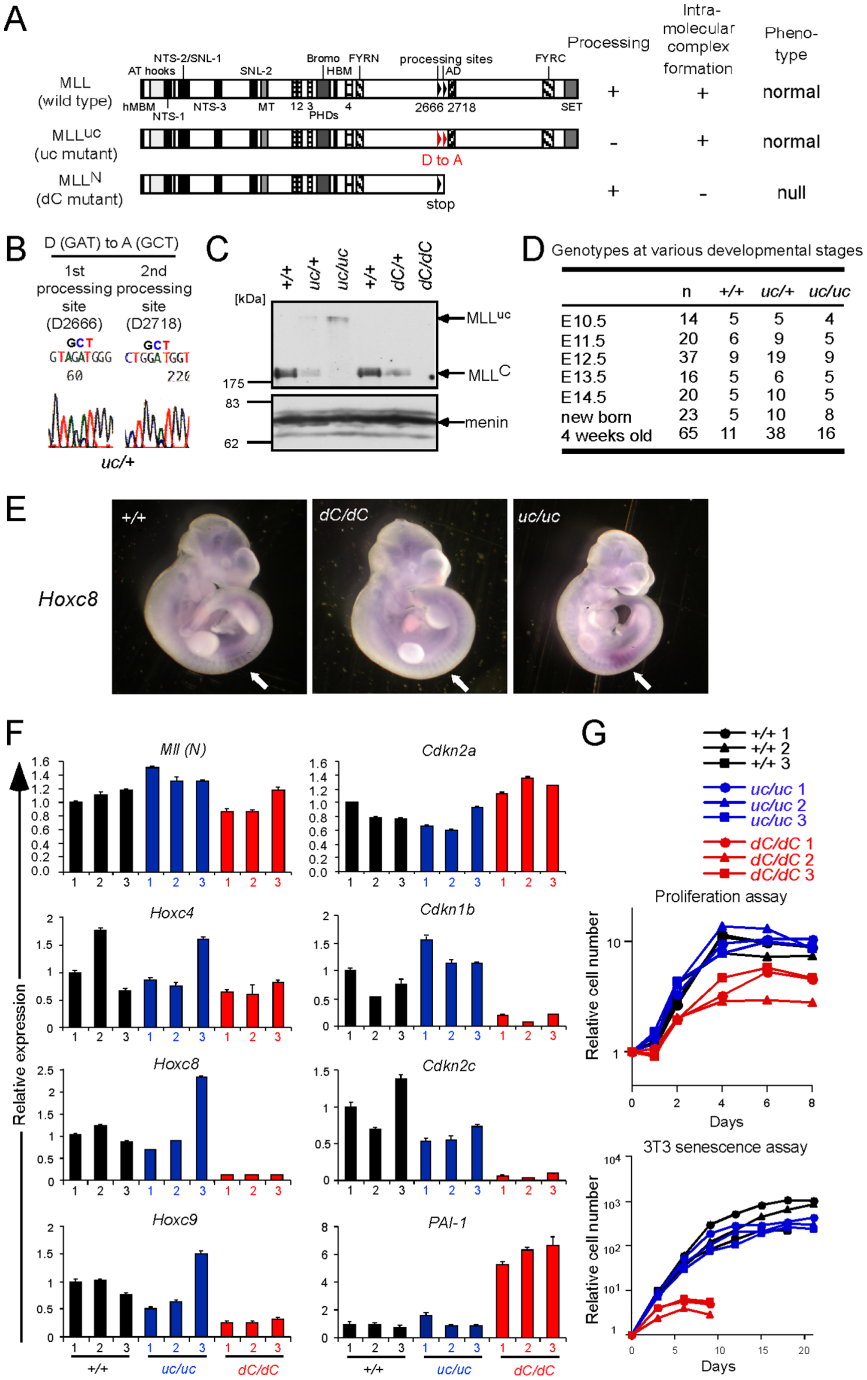
MLL processing is not required for MLL-dependent gene activation. A. Schematic structures of MLL proteins produced by *dC* and *uc* mutations. The characteristics of each mutant are shown on the right. The mutated processing sites are highlighted in red. B. Sequences at the processing sites of the PCR fragments amplified from genomic DNAs of the recombined ES cell clones. C. Expression of MLL proteins in embryos. Western blotting was performed on whole embryo extracts of various genotypes. MLL proteins were visualized by anti-MLL^C^ antibody. Anti-menin blot serves as a loading control. *uc/+*: *uc* heterozygous. *uc/uc*: *uc* homozygous. *dC/+*: *dC* heterozygous. *dC/ dC*: *dC* homozygous. D. Genotypes at various developmental stages. Viability of embryos was confirmed by presence of heart contractions. E. Expression of *Hoxc8* transcripts in E10.5 embryos. Whole mount in situ hybridization was performed using the *Hoxc8* probe *(Hoxc8*). Arrows indicate sites of target gene expression. Previously published data for the wild type and *dC* homozygous embryos [8] are shown here for comparison. F. Expression of various genes in mutant MEFs. Three independently established MEF lines of wild typ*e*, *uc* homozygous and *dC* homozygous genotypes were examined by RT-qPCR for genes indicated at the tops of respective panels. Relative expression levels (normalized to *Gapdh*) are expressed relative to those of wild type-1 arbitrarily set as 1. Previously published data for wild type and *dC* homozygous MEFs [8] obtained in the same experiment are included for comparison. Error bars represent the standard deviations of triplicate PCRs. G. Proliferative capacities of *uc* homozygous MEFs. Proliferation assay (top) and 3T3 senescence assay (bottom) were performed for three lines each of wild type, *uc* homozygous and *dC* homozygous genotypes at passage 3. Previously published data for wild type and *dC* homozygous MEFs [8] obtained in the same experiments are included for comparison.

To investigate the in vivo roles of proteolytic processing of MLL, we generated a knock-in mouse line (designated *uc*) with targeted germline mutations of the MLL processing sites. ES cells were engineered to contain an alanine substitution mutation at the critical aspartic acid residue in both processing sites (the murine counterparts of human D2666 and D2718) [8], [10] thereby expressing an uncleavable mutant of MLL (MLL^uc^) (Figure 3A). Genomic PCR followed by sequencing confirmed that recombined ES cells harbored the targeted allele (Figure 3B). Western blotting analysis confirmed expression of MLL^uc^ in *uc* mutant embryos (Figure 3C). *uc* homozygous mice were born at normal Mendelian ratios (Figure 3D) with no apparent anatomic/functional defects. *Hoxc8*, an MLL target gene [2], was properly expressed in *uc* homozygous embryos at E10.5, where *dC* homozygous embryos failed to maintain its expression [8] (Figure 3E). Thus, an inability to proteolytically process MLL does not compromise the developmental roles of MLL.

To further investigate the possible effect of MLL processing on MLL-dependent transcription, MEF cell lines of wild type, *uc* homozygous and *dC* homozygous genotypes were analyzed by RT-qPCR. In contrast to the comparable expression of *Mll* and *Hoxc4* mRNAs among all the cell lines, expression of MLL target genes including *Hoxc8*, *Hoxc9*, *Cdkn2c* and *Cdkn1b* [8], [22], [23] was substantially reduced in *dC* homozygous MEFs but unaffected in *uc* homozygous MEFs (Figure 3F). Although *Taspase I* knock-out MEFs, which exclusively express the unprocessed form of MLL, were reported to over-express *Cdkn2a*, *uc* homozygous MEFs expressed *Cdkn2a* at comparable levels to the wild type control, indicating that processing of MLL is not required for suppression of *Cdkn2a* expression. Furthermore, in contrast to a report by Takeda et al. [25], no severe growth retardation of *uc* homozygous MEFs was observed in proliferation assays and in 3T3 senescence assays, in the condition where *dC* homozygous MEFs displayed a premature senescence phenotype [8] (Figure 3G). Consistent with these results, *PAI-1* (also known as *Serpine-1*), a well-known senescence inducer [26], was highly expressed in *dC* homozygous MEFs, but expressed at normal levels in *uc* homozygous MEFs. Hence, processing of MLL is not required for MLL-dependent transcription in contrast to the severe compromise of MLL functions caused by the inability to self-associate.

### Lack of MLL processing does not affect steady state hematopoiesis

**Figure 4:**
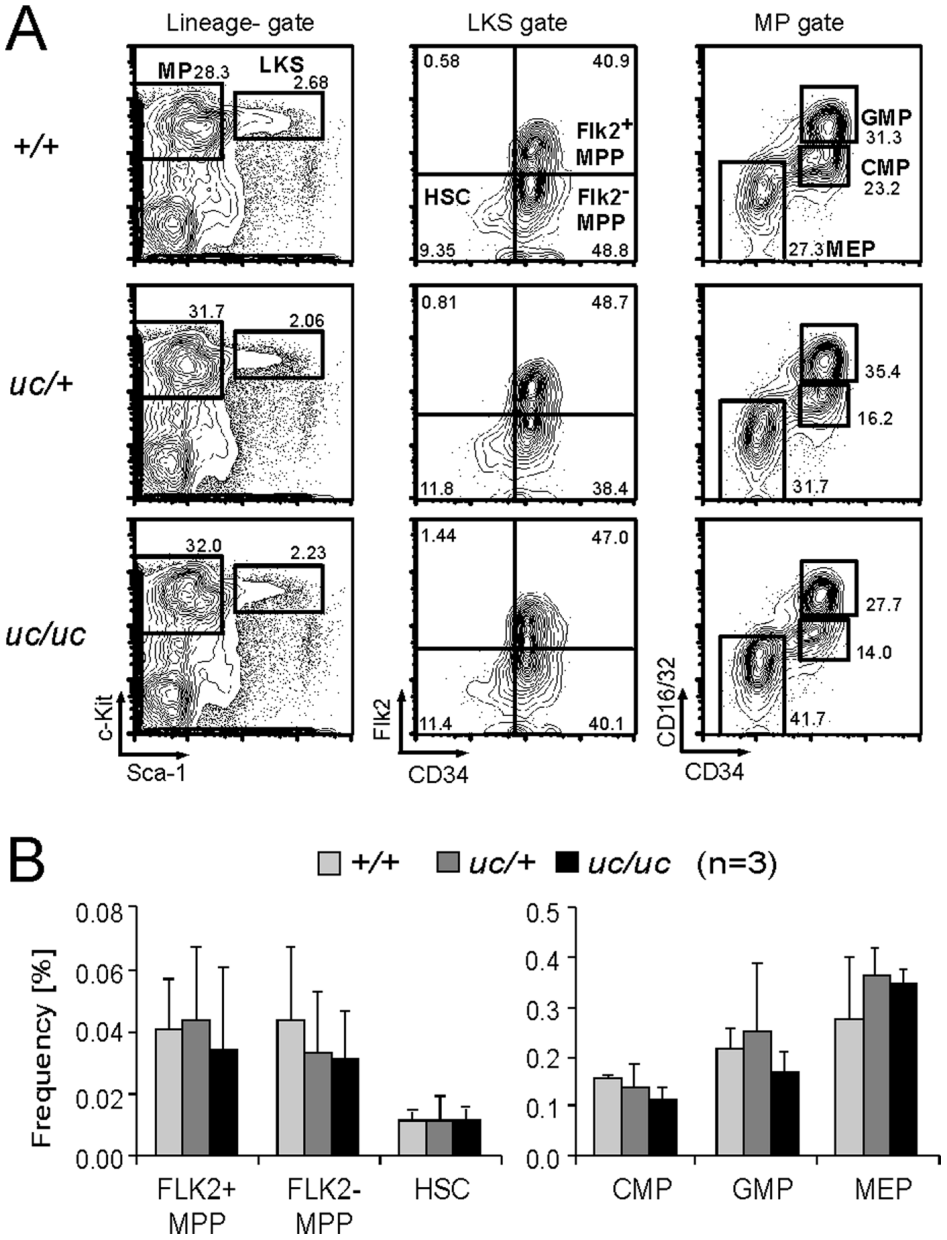
Processing of MLL is not required for adult hematopoiesis. A. Population analysis of Lin^−^cKit^+^Sca1^−^ (MP) and Lin^−^cKit^+^Sca1^+^ (LKS) compartments and their subcompartments in the BM of adult *uc* mutant mice. Lineage cocktail (anti-CD3, CD4, CD8, B220, TER119, Gr-1, Mac1/CD11b) was used to define lineage negative fractions. B. Frequencies of hematopoietic progenitors (Flk2^−^MPP, Flk2^+^MPP, CMP, GMP and MEP) and HSCs in *uc* mutant mice. Error bar represents standard deviations of three independent samples.

To investigate the role of MLL processing in hematopoiesis, we analyzed the hematopoietic compartments of adult *uc* mutant mice by flow cytometry (Figure 4A, 4B). HSCs, MPPs, and lineage-restricted progenitors including common myeloid progenitors (CMPs), granulocyte/monocyte progenitors (GMPs) and megakaryocyte/erythroid progenitors (MEPs) in bone marrow (BM) exhibited normal frequencies. Furthermore, T-cells in thymus, and B- cells in BM also exhibited normal compositions (data not shown). Thus, MLL processing is dispensable for steady-state hematopoiesis in adult mice.

### The uncleavable mutant of MLL is not constitutively active

**Figure 5:**
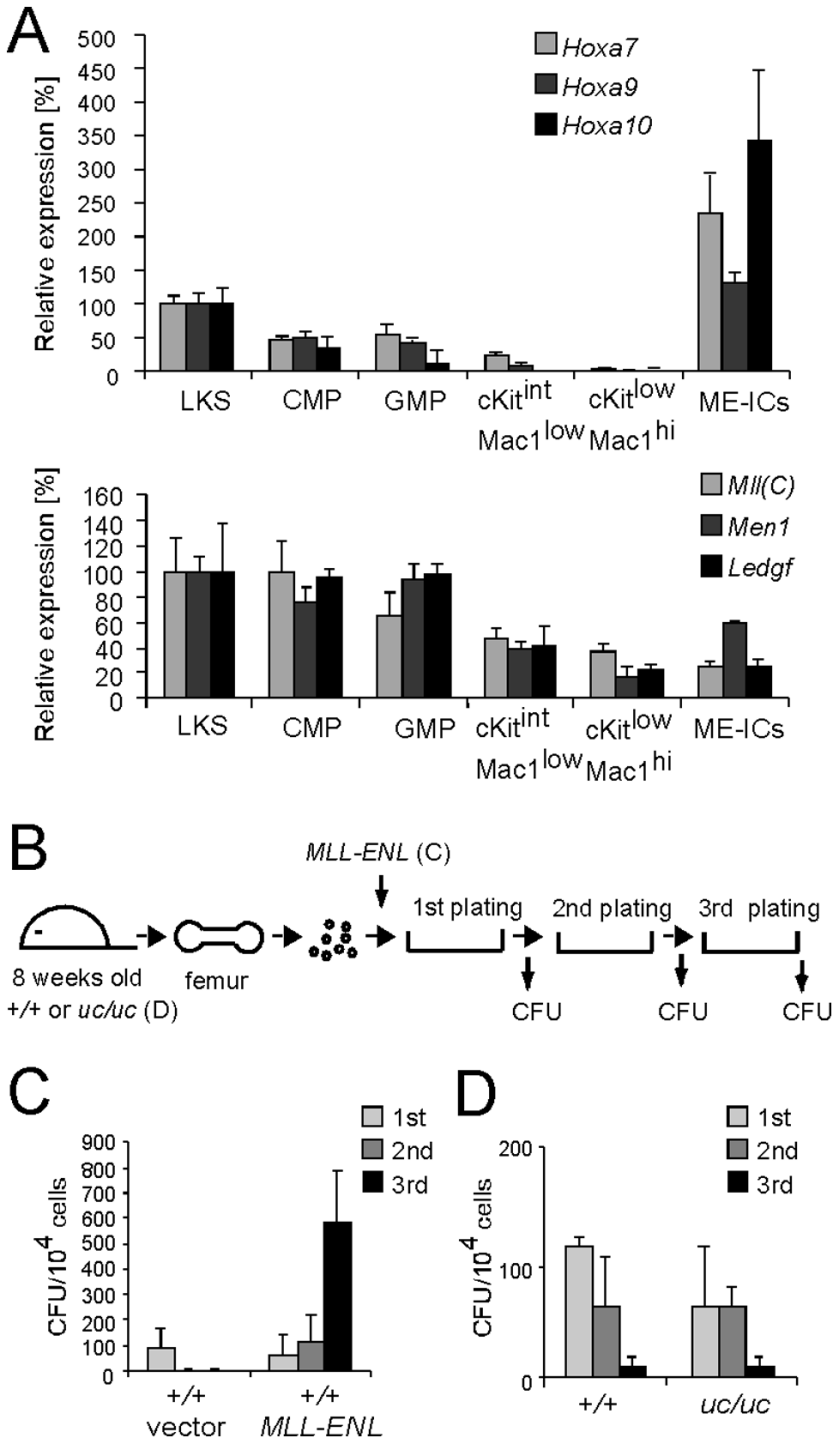
Lack of processing does not constitutively activate MLL. A. Expression of posterior *Hoxa* genes and MLL complex components during myeloid differentiation. Each population was isolated by cell sorting and analyzed by RT-qPCR. Relative expression levels (normalized to *Actb*) and expressed relative to those of LKS arbitrarily set as 100%. Error bars represent standard deviations of triplicate PCRs. B. Experimental scheme of myeloid progenitor serial replating assay. *MLL-ENL* was transduced into wild type myeloid progenitors in Figure 5C. Colony-forming activity of wild type and *uc* homozygous myeloid progenitors was analyzed without any gene transduction in Figure 5D. C. Clonogenic potentials of *MLL-ENL* (or vector)-transduced myeloid progenitors in myeloid progenitor serial replating assay. Transduced cells were cultured in semi-solid media and subjected to serial replating. CFUs per 10^4^ plated cells were enumerated after each round. Error bars represent standard deviations of three independent samples. D. Clonogenic potentials of myeloid progenitors derived from *uc* homozygous mice in myeloid progenitor serial replating assay. Error bars represent standard deviations of three independent samples.

MLL maintains *HOX* gene expression to promote expansion of hematopoietic progenitors during myeloid differentiation [3], [4]. Because *Hox* gene expression progressively declines as cells differentiate [12], [27] (Figure 5A, upper panel), the transcriptional activity of MLL is presumed to decline in parallel. Transcripts for essential components of the MLL complex (MLL itself, menin and LEDGF) are also down-regulated as cells differentiate (Figure 5A, lower panel). However, their declining expression levels were not quantitatively in accord with those of *Hox* genes. Noticeably, their expression levels in differentiated cells (ckit^low^Mac1^high^) were similar to the levels present in *MLL-ENL*-transformed cells, whose growth is critically dependent on menin and LEDGF [28], [29], [30]. These data suggest that the decline of *Hox* gene expression during differentiation is not entirely due to the decrease of MLL complex components at the mRNA level; rather, a post-transcriptional regulatory mechanism might mediate down-regulation of MLL-dependent transcription. We hypothesized that dissociation of the MLL^C^ subunit may occur during differentiation to extinguish MLL function, whereas uncleavable MLL might function as the constitutively active form. In this scenario, myeloid progenitors derived from *uc* homozygous mice should exhibit enhanced clonogenicity in vitro similar to that induced by MLL-ENL, which behaves as the constitutively active form that aberrantly maintains *Hoxa9* expression and promotes proliferation (Figure 5B, 5C). However, *uc* homozygous myeloid progenitors did not display enhanced replating activity compared to the wild type control (Figure 5B, 5D). Thus, lack of processing does not render MLL constitutively active in hematopoietic progenitors.

### MLL processing is not required for oncogene-dependent proliferation of myeloid progenitors

**Figure 6:**
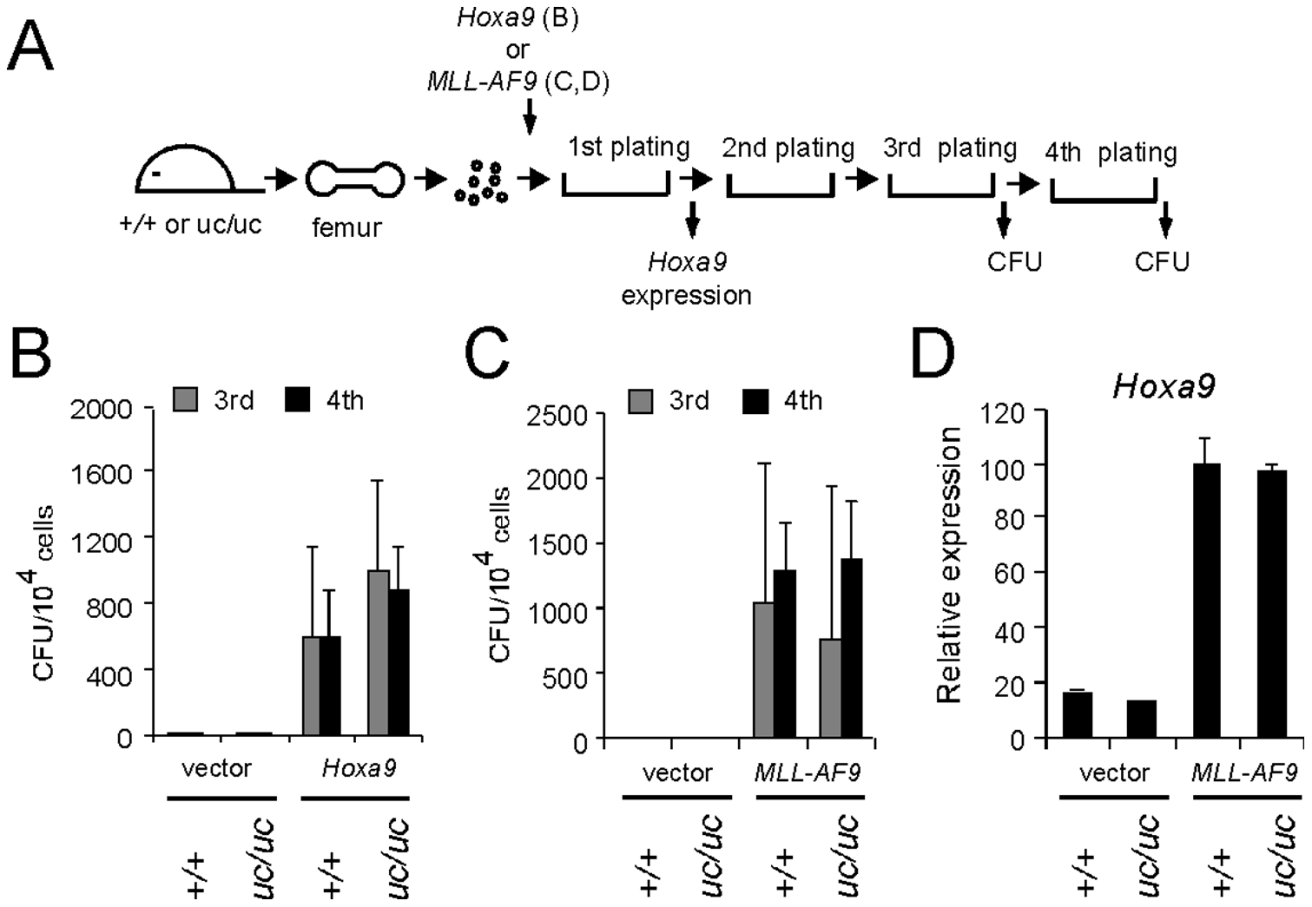
MLL processing is not required for proliferation of oncogene-transformed myeloid progenitors. A. Experimental scheme of myeloid progenitor serial replating assay. *Hoxa9* was transduced in Figure 6B and *MLL-AF9* in Figure 6C and 6D. The time points at which colony forming units and *Hoxa9* expression were measured are shown. B. Clonogenic potentials of *Hoxa9*-transformed myeloid progenitors of wild type or *uc* homozygous mutant origin. CFUs per 10^4^ plated cells were enumerated at third and fourth round. Error bars represent standard deviations of three independent samples. C. Clonogenic potentials of *MLL-AF9*-transformed myeloid progenitors of wild type or *uc* homozygous mutant origin. CFUs per 10^4^ plated cells were enumerated at third and fourth round. Error bars represent standard deviations of three independent samples. D. *Hoxa9* expression in the first round colonies of *MLL-AF9*-transformed cells. Relative expression levels (normalized to *Gapdh*) and expressed relative to those of *MLL-AF9*-transformed wild type progenitors arbitrarily set as 100%. Error bars represent standard deviations of triplicate PCRs.

To examine whether MLL processing is required for the enhanced proliferation of hematopoietic progenitors induced by oncogenes, we transduced myeloid progenitors derived from *uc* homozygous mice with *Hoxa9* expression vector and analyzed their serial replating activity (Figure 6A). Despite the lack of processing, *Hoxa9*-transformed *uc* homozygous myeloid progenitors exhibited clonogenicity comparable to the wild type control (Figure 6B). These results demonstrate that MLL processing is not required for proliferation of oncogene-transformed myeloid progenitors.

It has been reported that the wild type MLL protein is required for leukemic transformation by the *MLL-AF9* oncogene [28]. This notion presumes that the function of MLL must be preserved for MLL-AF9 to activate *Hoxa9* expression and transform myeloid progenitors. MLL-AF9 successfully transformed *uc* homozygous myeloid progenitors (Figure 6C) and activated *Hoxa9* expression (Figure 6D) in a serial replating assay (Figure 6A). Hence, the uncleavable form of MLL is equivalently functional to the processed form of MLL in MLL-AF9-transformed myeloid progenitors.

### MLL processing is not required for self-renewal of HSCs in vivo

**Figure 7:**
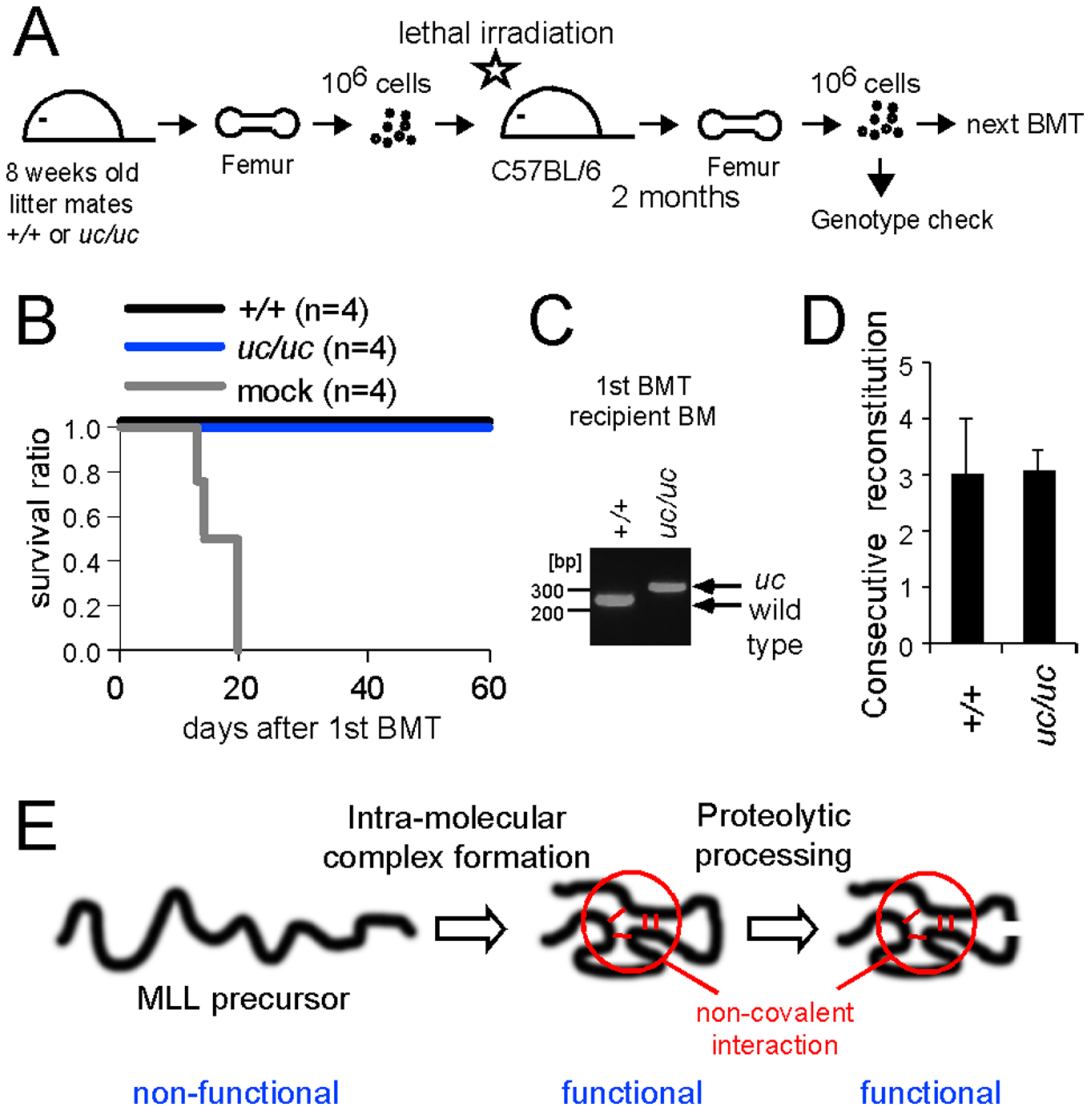
MLL processing is not required for self-renewal of hematopoietic stem cells. A. Experimental scheme of serial reconstitution assay. The time point at which the genotype of hematopoietic cells was examined is indicated. B. Survival of lethally irradiated mice transplanted with BM cells derived from adult *uc* homozygous mice and their littermate wild type control. Mock control mice die two to three weeks after irradiation. C. Genotype of reconstituted hematopoietic cells in the BM of recipient mice. The BM cells were prepared from femurs and subjected to genotyping PCRs. D. Reconstitution potentials of the donor cells before eventual exhaustion of HSCs. Recipient mice that survived for 60 days after transplantation were subjected to the next round of bone marrow transplantation (BMT). Average times of successful reconstitution were expressed with error bars that represent standard deviations of nine independent samples. E. Model of MLL protein maturation.

Previous studies have shown that MLL serves an important role in self-renewal of HSCs and therefore is necessary to reconstitute the hematopoietic system in vivo [3], [7], [8] (Figure 2F). To examine the role of MLL processing in self-renewal, we performed serial in vivo reconstitution assays using wild type and *uc* homozygous donor cells derived from littermate mice (Figure 7A). *uc* homozygous BM cells successfully reconstituted the hematopoietic system as well as the wild type control (Figure 7B, 7C). Serial reconstitution assays showed that *uc* homozygous HSCs and the wild type control consecutively reconstituted three times on average before the eventual depletion of HSCs (Figure 7D), demonstrating that *uc* homozygous HSCs retain equivalent reconstituting potentials to the wild type control. These results indicate that MLL processing is not required for self-renewal of HSCs in vivo.

## Discussion

In this study, we generated mouse mutant alleles that effectively inhibit intra-molecular complex formation and MLL processing, respectively, to address the in vivo roles of these two maturation steps. Loss of intra-molecular interaction had devastating effects and manifested a null phenotype, demonstrating that intra-molecular interaction of MLLN and MLLC is essential for MLL functions. These results indicate that loss of intra-molecular interaction exposes the FYRN domain, which triggers degradation and leads to loss-of-function of MLL as we previously reported [8]. On the other hand, in spite of the evolutionary conservation of the processing sites, loss of processing caused no measurable defects, indicating that processing is mostly dispensable for MLL-dependent functions. Furthermore, *uc* homozygous cells have normal proliferation capacity, demonstrated by proliferation assay of MEFs, serial replating assay of oncogene-transformed hematopoietic progenitors, and serial in vivo reconstitution assay. Therefore, our results do not support the previously suggested essential role of MLL processing in cell cycle progression. Taken together, MLL processing is not required for its function unlike many other bioactive peptides that become activated by proteolytic cleavage, whereas intra-molecular complex formation is an essential step in MLL maturation. Because cleavage occurs to the MLL mutant proteins that are incapable of intra-molecular interaction, the intra-molecular interaction itself is not a prerequisite for Taspase 1-dependent cleavage [8]. However, if cleavage occurs before the intra-molecular interaction, MLL fragments would dissociate from each other and be subjected to degradation. Hence, we propose a model in which intra-molecular interaction takes place first, followed by Taspase 1-dependent cleavage in the proper maturation process of MLL (Figure 7E).

Previous analysis of fetal and adult hematopoiesis showed that MLL is required for sufficient proliferative expansion of hematopoietic progenitors [3], [4], [7], [8]. During myeloid differentiation, MLL maintains expression of posterior *Hoxa* genes [3], [4], which are highly expressed in HSCs/MPPs and progressively down-regulated in more differentiated progenitors [12], [27] (Figure 4A). Posterior *Hoxa* genes are required to sufficiently promote proliferation of undifferentiated hematopoietic progenitors [5], [6], [12]. Hence, MLL-dependent *HOX* gene expression is necessary to maintain appropriate pool sizes of HSCs and immature progenitors. Our in vivo analyses of *uc* homozygous and *de* homozygous mutant mice show that intra-molecular complex formation but not MLL processing is required for proper expansion of hematopoietic progenitors. MLL fusion proteins generated by chromosomal translocations constitutively activate posterior *HOXA* genes to cause leukemia and therefore those mutations are defined as gain-of-function. Our analysis of *de* homozygous MEFs showed that loss of intra-molecular complex formation results in degradation of MLL proteins and impaired expression of MLL target genes. Thus, although deletion of exon 11 was originally found in ALL [21], this mutation is defined as loss-of-function, suggesting that this mutation likely contributes to leukemogenesis through different mechanisms from MLL fusion-dependent leukemogenesis.

MLL fragments generated by proteolytic processing associate with each other by non-covalent interaction, which potentially allows conditional dissociation of the MLL holocomplex. In accord with this hypothesis, genome wide ChIP analysis of Drosophila embryos showed that trithorax (TRX) protein fragments generated by processing differentially associate with the Drosophila genome [31]. Therefore, it was thought that MLL^C^ might dissociate from MLL^N^ in a context-dependent manner to become functionally inactive. In this scenario, the unprocessed mutant protein might function as the constitutively active form. However, unlike MLL fusion proteins, the uncleavable mutant did not enhance serial replating activity of hematopoietic progenitors (Figure 5D), indicating that conditional dissociation of MLL^C^ is not the major mechanism for extinguishing MLL activity during myeloid differentiation.

It has been proposed that MLL processing might be important for maintaining expression of the Antenapedia complex (ANT-C) genes. This concept was originated by the study of a Drosophila TRX mutant (trx^E3^) that contains an internal deletion of amino acids encompassing the processing site and therefore exclusively expresses an uncleavable mutant protein [32], [33]. trx^E3^ exhibits mildly reduced ANT-C expression in late stages and normal bithorax complex (BX-C) expression, whereas the TRX null mutant (trx^B11^) displays mildly decreased expression of ANT-C genes in late embryonic development and severely decreased expression of BX-C genes in early embryonic development. Thus, it was hypothesized that TRX processing might be required specifically for late ANT-C expression in fly embryonic development. However, our current results show that *uc* homozygous mice have no developmental defects. Furthermore, expression of *Hoxc4*, which is a member of the ANT-C genes, in *uc* homozygous MEFs was comparable to the wild type control (Figure 3F). Expression of *Hoxc8*, which is a member of the BX-C genes, was severely decreased in *dC* homozygous MEFs, but unaffected in *uc* homozygous MEFs. Thus, our results indicate that the role of MLL processing on *HOX* gene expression is minor, if any, in mammalian development.

Taspase 1 knock out mice demonstrate various defects including smaller body size, reduced MEF proliferation, and skeletal structural anomalies [25]. More specifically, Taspase 1 deficiency causes upregulation of CDK inhibitors such as p16 and ARF (the products of the *Cdkn2a* gene) to inhibit proliferation of MEFs. However, *uc* homozygous mice were born with normal body size, and *uc* homozygous MEFs did not display severely altered expression of *Cdkn2a* (Figure 3F) nor manifested severe proliferation defects (Figure 3G) in contrast to a previous study that also analyzed mice engineered to express an uncleavable MLL protein [25]. The basis for these differences between the two independently generated mouse lines is unclear since detailed description of the mice generated by Takeda et al. was not reported [25], but may be due to differences in genetic backgrounds of the ES cells or the targeting vector. Nevertheless, our results indicate that MLL processing does not serve rate-limiting roles for proliferation and suggest that the growth defects caused by Taspase 1-deficiency may be attributed to other substrates such as MLL2 [25] or TFIIA [34].

Taken together, our results indicate that MLL processing has no biological roles in development, hematopoiesis and proliferation, whereas intra-molecular interaction of MLL is essential in all of those circumstances. However, evolutionary conservation of the processing sites suggests that MLL processing has some biological roles. It may be important under other circumstances not tested in this study such as stress conditions and immunity.

## Conclusions

In the current study, we examined the in vivo roles of intra-molecular interaction and proteolytic processing of MLL in various assays. Loss of intra-molecular interaction caused loss-of-MLL function, whereas loss of processing caused no detectable functional alterations. These results unequivocally demonstrate that formation of an intra-molecular complex is, but the processing is not, required for MLL-dependent gene activation and cell proliferation.
